# Self-compassion and parenting efficacy among mothers who are breast cancer survivors: Implications for psychological distress

**DOI:** 10.1177/13591053231222162

**Published:** 2024-01-23

**Authors:** Carissa Nadia Kuswanto, Lesley Stafford, Penelope Schofield, Jessica Sharp

**Affiliations:** 1 Swinburne University of Technology, Australia; 2 University of Melbourne, Australia; 3 Peter MacCallum Cancer Centre, Australia

**Keywords:** anxiety, breast cancer, depression, mothers, parenting efficacy, self-compassion, stress

## Abstract

Mothers who are breast cancer survivors may experience psychological distress in relation to diminished parenting efficacy. Self-compassion may protect mothers from psychological distress, yet little is known about self-compassion in this population. The extent to which self-warmth (self-kindness, mindfulness and sense of common humanity) and self-coldness (self-judgement, isolation and over-identification) dimensions of self-compassion moderate parenting efficacy in predicting depression, anxiety and stress was examined in a sample of 95 mothers who were breast cancer survivors. Independently, poorer parenting efficacy was associated with more depression and stress symptoms. Within regression models, self-coldness was a direct predictor of depression, anxiety and stress, while self-warmth moderated the relationship between parenting efficacy and stress. Self-warmth presents as a potential protective factor for stress associated with poor parenting efficacy, while self-coldness is a potential direct risk factor for psychological distress. Mothers who are breast cancer survivors may benefit from self-compassion focused psychosocial interventions.

## Introduction

Many mothers diagnosed with cancer experience a range of challenges as they juggle caring for themselves and their children while managing cancer symptoms and treatment side effects (Kuswanto et al., 2018; Tavares et al., 2018). Symptoms of cancer and the side effects of treatment often interfere with their perceived ability to be a ‘good’ mother in providing physical, emotional and psychological care for their children (Fisher and O’Connor, 2012; Fitch et al., 1999; Park et al., 2017; Semple and McCance, 2010). Some mothers also report that their illness affects their children’s wellbeing and the dynamics of the mother-child relationship (Tavares et al., 2018). Fathers and extended family are often seen as less-than-ideal substitutes for mothers (Bell and Ristovski-Slijepcevic, 2011; Park et al., 2017) and mothers who struggle with parenting duties may nevertheless feel the pressure of cultural and societal demands for them to continue in the parenting role as before, despite their cancer diagnosis (Elmberger et al., 2000; Fisher and O’Connor, 2012; Semple and McCance, 2010). Given the challenges of undertaking their mothering roles since receiving a cancer diagnosis, and the possible negative consequences on their children and family that follow, it is not surprising that mothers report disruption to their sense of identity and role within the family (Fisher and O’Connor, 2012; Fitch et al., 1999; Mackenzie, 2014; Mazaheri et al., 2021).

Parenting efficacy is a caregiver’s confidence in their ability to successfully raise their children and engage in the behaviours expected in their role as parents (Albanese et al., 2019; Jones and Prinz, 2005). Mothers’ experiences of identity disruption following a cancer diagnosis appear to intertwine with their sense of parenting efficacy and perceived inability to meet the emotional, physical and social needs of their children (Fisher and O’Connor, 2012; Mazaheri et al., 2021). Research has shown parenting efficacy to decline following a cancer diagnosis (Moore et al., 2015) and recent systematic reviews of both qualitative and quantitative research reveal that mothers who report diminished parenting efficacy may experience guilt, shame and considerable psychological distress such as depression, anxiety and stress (Kuswanto et al., 2018; Tavares et al., 2018). Unfortunately, targeted psychosocial interventions for mothers with cancer are scarce, which further highlights the importance of exploring strategies to address psychological distress associated with parenting efficacy in this population.

Both theory and research indicate self-compassion may present a promising point of intervention to protect against psychological distress among mothers who are breast cancer survivors. Self-compassion is the practice of being kind and understanding towards oneself in instances of suffering or pain (Neff, 2003a, 2003b). Three theoretical facets of self-compassion are presented: self-kindness versus self-judgement, common humanity versus isolation and mindfulness versus over-identification (Neff, 2003b). Common humanity represents an awareness and acknowledgement that one’s suffering does not occur in isolation, but is inherent to life circumstances (Neff, 2003b; Van Dam et al., 2011). Mindfulness is holding one’s painful thoughts and feelings in a balanced frame of mind rather than suppressing them or over-identifying with them (Neff, 2003b), allowing one to witness the experience of suffering in an objective light (Reyes, 2012). Those who engage in self-kindness are less likely to engage in self-criticism, self-blaming and judgemental rumination in response to perceived shortfalls (Raes, 2010).

Research has demonstrated that greater self-compassion is associated with lower depressive and anxiety symptoms, and increased resilience to stress, in both clinical and non-clinical populations (Diedrich et al., 2014; Krieger et al., 2013; MacBeth and Gumley, 2012), and with higher quality of life and lower levels of depressive, anxiety and stress symptoms in cancer patients and survivors (Arambasic et al., 2019; Pinto-Gouveia et al., 2014; Przezdziecki et al., 2013). Furthermore, self-compassion has recently been shown to moderate the relationship between perceived stress and self-care behaviours in women with breast cancer, suggesting self-compassion may increase the ability to manage health-related behaviours and buffer the effect of stress during difficult periods of illness (Abdollahi et al., 2020). Though the role of self-compassion has not yet been explored in mothers who may face additional parenting challenges due to their cancer diagnosis, these findings suggest enhancing self-compassion may be an appropriate intervention for this population.

Most self-compassion research has used the Self-Compassion Scale (SCS; Neff, 2003a). Self-compassion is operationalised in the SCS as a unitary construct consisting of three continuous dimensions ranging from positive to negative (e.g. from self-kindness to self-judgement), and the total SCS score is obtained through summing the scores from the positive dimension and the inverse scores from the negative dimension (Neff, 2003a, 2016). However, there has been some criticism regarding the use of the SCS total score as an overall indicator of self-compassion (Costa et al., 2016; López et al., 2015; Muris, 2016; Phillips and Ferguson, 2013). Instead, recommendations for a two-factor model of self-compassion have emerged; one being ‘self-warmth’, which encapsulates the three positive subscales (self-kindness, sense of common humanity and mindfulness), and the other being ‘self-coldness’, consisting of the three negative subscales (self-judgement, isolation and over-identification) of the SCS (Brophy et al., 2020; Costa et al., 2016; López et al., 2015; Muris, 2016; Phillips and Ferguson, 2013). Phillips and Ferguson (2013) demonstrated these two dimensions of the SCS make unique predictive contributions to psychological outcomes. Specifically, self-warmth predicted positive affect whilst self-coldness predicted negative affect, indicating self-warmth and self-coldness should be considered separately in relation to psychological distress.

Mothers with cancer symptoms and treatment side effects may experience poorer parenting efficacy (Cessna et al., 2016; Moore et al., 2015) and this may be associated with symptoms of psychological distress, including anxiety, depression or stress, in response to their perceived shortcomings (Kuswanto et al., 2018; Moore et al., 2015). Self-compassion reflects treating oneself kindly in the face of struggles and failures (Allen and Leary, 2010). It is plausible therefore that mothers who engage in more self-warmth may be less likely to experience symptoms of psychological distress in association with poor parenting efficacy because they tend to (1) be more likely to have an honest and non-judgemental understanding of their own parenting struggles, (2) not avoid nor disconnect from pain and suffering and (3) forgive their failings and respect themselves as limited and imperfect parents to their children. On the contrary, mothers who engage in self-coldness may be more likely to experience symptoms of psychological distress associated with poor parenting efficacy because they are more likely to (1) ruminate and be self-judgemental of their parenting failings, (2) have difficulties in acknowledging their feelings and (3) feel isolated within their own sufferings.

Our aim was to explore these potential roles of self-warmth and self-coldness in moderating the relationship between parenting efficacy and symptoms of psychological distress in mothers who are breast cancer survivors. Overall, we expected poorer parenting efficacy to be associated with higher levels of psychological distress. However, we predicted that this association between parenting efficacy and psychological distress would vary depending on mothers’ levels of self-warmth and self-coldness. It was hypothesised that the relationship between parenting efficacy and psychological distress symptoms would be weaker for mothers high in self-warmth compared to those low in self-warmth (buffering hypothesis). We further hypothesised that the relationship between parenting efficacy and psychological distress symptoms would be stronger for mothers high in self-coldness than for those low in self-coldness (synergistic hypothesis). These hypotheses were examined for three different facets of psychological distress: depression, anxiety and stress.

## Method

### Setting and participants

Mothers who were breast cancer survivors were recruited from the Breast Cancer Network Australia, an online network providing information and support for Australians affected by breast cancer. Inclusion criteria were being 18 years or older, being a mother at the time of diagnosis and being able to complete the questionnaire in English. The cross-sectional data was collected anonymously via an online questionnaire, and written informed consent was provided at the commencement of the questionnaire. Ethics approval was obtained from Swinburne University Human Research Ethics Committee (SHR Project 2017/008).

### Measures

#### Demographic and clinical information

Demographic and clinical information collected included age, relationship status, age and number of children, highest education level, occupation and clinical details (disease stage, type of treatment, time since receiving the diagnosis and time since initial surgery).

#### Depression, anxiety and stress

The three psychological distress outcome variables, depression, anxiety and stress were measured using the 21-item DASS-21 scale (Lovibond and Lovibond, 1995). Seven items measure each dimension, and items are rated using a 4-point Likert scale, ranging from 0 (*did not apply to me at all*) to 3 (*applied to me very much, or most of the time*). Scores of at least 10 for depression (DASS-D), 8 for anxiety (DASS-A) and 15 for stress (DASS-S) indicate clinical levels of distress for each subscale. In accordance with the DASS manual (Lovibond and Lovibond, 1995), DASS21 subscale scores were multiplied by two to convert them to full-subscale scores. The DASS-21 has good reliability and validity, and is moderately sensitive to change (Page et al., 2007). For our study, the DASS subscales had high internal reliability (Cronbach’s α = 0.91 for depression, α = 0.81 for anxiety and α = 0.86 for stress).

#### Cancer-related parenting self efficacy

Parenting efficacy was assessed with the Cancer-Related Parenting Self Efficacy scale (CaPSE; Cessna et al., 2016), which comprises 24 items asking parents to rate how confident they feel in their ability to perform tasks that might be particularly difficult for parents who may have certain limitations due to cancer and its treatment side effects. With the stem phrase: ‘I am confident about my ability to. . .’, items assess respondents’ confidence for example to ‘. . .meet the emotional needs of my child’, ‘. . . be there physically when my child needs me’ and ‘. . .meet the expectations I have for myself as a parent’. Items are rated using a 6-point Likert scale, ranging 1 (*strongly disagree*) to 6 (*strongly agree*). In the current study, the CaPSE had high internal reliability (α = 0.95).

#### Self-compassion

The 26-item Self Compassion Scale (SCS; Neff, 2003a) comprises six subscales: three positive (self-kindness, common humanity and mindfulness), and three negative (self-judgement, isolation and over-identification). Items are rated using a 5-point Likert scale, ranging from 1 (*almost never*) to 5 (*almost always*). Mean scores are obtained for the three positive dimensions to indicate *self-warmth* and for the three negative dimensions to indicate *self-coldness*. The total SCS has high internal reliability (α = 0.93) in prior research (Neff, 2003a), and self-coldness (α = 0.93) and self-warmth (α = 0.90) also had high internal reliability in the current study.

### Data analysis

Descriptive statistics, Pearson’s correlation and hierarchical linear regressions were conducted using the Statistical Package for Social Sciences (SPSS v28). Bivariate correlations between the clinical and demographic variables, primary predictors (parenting efficacy, self-warmth and self-coldness) and outcome variables (depression, anxiety and stress) were examined. Non-predictors with significant correlations (*p* < 0.05) with depression, anxiety or stress were used as covariates in the main analysis.

The moderation hypotheses were investigated using hierarchical linear regression in PROCESS v4.1 Model 2 which allows two interactions in the model. The predictor was parenting efficacy (X) while self-warmth (W) and self-coldness (Z) were entered as moderators. The predictor and moderators were centred for the computation of the interaction terms (self-warmth × parenting efficacy and self-coldness × parenting efficacy) as per Aiken and West (1991). The analysis was repeated for each outcome variable (Y): depression, anxiety and stress. Relevant covariates were added to the model. InterActive (https://connorjmccabe.shinyapps.io/interactive/), an open-source analysis and data-visualisation application was used for the interaction plot. For each simple slope (e.g. slope at ±1SD of the moderator), InterActive displays the 95% CI for the predicted value of *Y* conditional on each observed value of *X* and the observed data underlying an interaction, in keeping with best practice for the visual display of interactions (McCabe et al., 2018).

From a total of 146 participants who provided informed consent, 13 participants consented but provided no data, and 38 participants were excluded because of incomplete data. A total of 95 participants were included in the study. Power analyses using G*Power 3.1 (Faul et al., 2007) showed that the sample size (*N* = 95) was adequate to detect moderate effects for the models for depression and stress with five predictors (*f*^2^ = 0.15, *p* ⩽ 0.05, power = 0.82), but less so for anxiety which included five additional covariates (*f*^2^ = 0.15, *p* ⩽ 0.05, power = 0.68). Accordingly, the analysis predicting anxiety was also performed without the covariates for comparison.

## Results

Supplemental Table 1 summarises the characteristics of the sample and the descriptive statistics for the measures. Participants’ mean age was 50.92 (SD = 6.63) and they had on average 2.23 children (SD = 0.81) ranging from 1 child to 6 children each. All participants had undergone surgery (mastectomy or lumpectomy), and around half (53.7%) were currently receiving hormonal therapy. Means, standard deviations and observed ranges for depression, anxiety and stress scores are also presented in Supplemental Table 1. In addition, notable percentages of participants reported higher than normal levels of distress, from mild to extremely severe (depression: 26.3%, anxiety: 17.9% and stress: 27.4%).

The correlations are shown in Supplemental Table 2. Parenting efficacy was significantly negatively correlated with both depression and stress, though not with anxiety. Similarly, self-warmth was significantly negatively correlated with depression and stress, though not with anxiety. Self-coldness was significantly positively correlated with depression, anxiety and stress. Parenting efficacy was significantly negatively correlated with self-coldness and significantly positively correlated with self-warmth. Covariates age of mother and age of youngest child, number of children, time since receiving cancer diagnosis and employment status were significantly correlated with anxiety.

Table 1 summarises the results of the model analyses predicting depression, anxiety and stress.

**Table 1.** Parenting efficacy and self compassion predicting depression, anxiety and stress in mothers who are breast cancer survivors.

Outcome	*B*	SE	*F*	*R* ^2^	Δ*R*^2^
*Depression*					
*Model*			6.49***	0.27***	0.01
Parenting efficacy	−0.09	0.07			
Self-warmth	0.02	1.20			
**Self-coldness**	**4.02** ***	0.95			
Parenting efficacy × Self-warmth	0.08	0.12			
Parenting efficacy × Self-coldness	0.05	0.10			
*Anxiety*					
*Model*			3.05*	0.15*	0.01
Parenting efficacy	−0.001	0.06			
Self-warmth	0.10	0.06			
**Self-coldness**	**2.44** ***	0.78			
Parenting efficacy × Self-warmth	0.08	0.09			
Parenting efficacy × Self-coldness	0.002	0.08			
*Anxiety*					
*Model*			4.52***	0.35***	0.01
Age of mother	−0.12	0.12			
**Age of youngest child**	**0.27** *	0.12			
**Number of children**	**2.49** ***	0.70			
Employment status	−1.56	1.31			
Time since diagnosis	−0.01	0.01			
Parenting efficacy	0.02	0.05			
Self-warmth	0.14	0.91			
**Self-coldness**	**2.76** ***	0.74			
Parenting efficacy × Self-warmth	0.08	0.09			
Parenting efficacy × Self-coldness	0.002	0.08			
*Stress*					
*Model*			13.43***	0.43***	0.06*
Parenting efficacy	−0.11	0.06			
Self-warmth	−0.73	1.08			
**Self-coldness**	**4.49** ***	0.86			
**Parenting efficacy × Self-warmth**	**0.24** *	0.11			
Parenting efficacy × Self-coldness	0.01	0.09			

Model statistics are shown for each step. Coefficient statistics are shown for the final step. Bold indicates significant predictors.

* *p* < 0.05. ***p < 0.001.

Within the models, parenting efficacy was not a significant direct predictor of depression, anxiety or stress. No significant interactions were found between parenting efficacy and self-coldness in predicting depression, anxiety or stress. However, consistent with the correlations, there were significant positive direct relationships between self-coldness and depression, anxiety and stress. In contrast, there were no direct relationships between self-warmth and depression, anxiety or stress. As seen for self-coldness, there were no significant interactions between parenting efficacy and self-warmth in predicting depression or anxiety. Depression was predicted by self-coldness, and anxiety was predicted by self-coldness, age of youngest child and number of children. The analysis was repeated for anxiety without the covariates and there were no substantive differences to the results for parenting efficacy, self-warmth or self-coldness, suggesting the lack of interaction effects was not due to reduced power from the inclusion of covariates.

**Figure 1.**
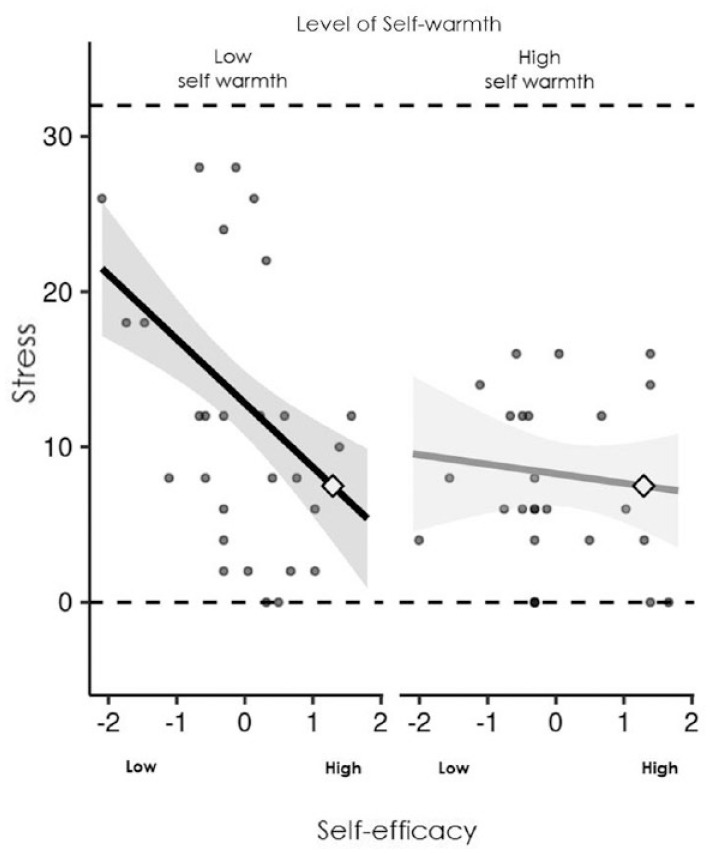
Parenting efficacy predicting stress at high (+1SD) and low (−1SD) self-warmth. Shaded area: 95% confidence region for slope; grey circles: observed data; dashed horizontal lines: maximum and minimum values of the outcome (stress); diamond: crossover point.

Stress was also predicted by self-coldness, however there was also a significant interaction effect for parenting efficacy and self-warmth in predicting stress, which accounted for an additional 6% of variance in stress. The relationships between parenting efficacy and stress at low (−1SD below the mean) and high (+1SD above the mean) levels of self-warmth are presented in Figure 1. There was a significant negative association between parenting efficacy and stress for low levels of self-warmth, *B* = −4.13, 95% CI [−6.14, −2.12], but no significant association between parenting efficacy and stress for high levels of self-warmth, *B* = −0.61, 95% CI [−2.57, 1.35].

## Discussion

Our aim was to explore the potential roles of self-warmth and self-coldness in moderating the relationship between parenting efficacy and symptoms of psychological distress in mothers who are breast cancer survivors. As suggested, mothers who are breast cancer survivors may experience more psychological distress (specifically depression and stress symptoms) in association with poorer parenting efficacy. Importantly, our findings demonstrated a buffering effect, whereby parenting efficacy was moderated by self-warmth in predicting stress in mothers diagnosed with breast cancer. Mothers with low levels of self-warmth (i.e. self-kindness, mindfulness and common humanity) experienced more stress in association with poor parenting efficacy, but mothers with high levels of self-warmth did not. Whilst self-coldness did not moderate the relationships between parenting efficacy and depression, anxiety or stress as had been predicted, our findings of direct relationships between self-coldness and all three psychological distress variables is consistent with emerging research indicating self-coldness is a key predictor of psychological distress (Brenner et al., 2017; Brophy et al., 2020; Muris et al., 2019; Muris and Petrocchi, 2017). Furthermore, our findings that self-warmth functioned as a moderator in predicting psychological distress, whereas self-coldness was a strong direct predictor, further demonstrate the value of investigating the two dimensions of self-compassion individually rather than as a unitary construct.

Past research revealed that cancer diagnosis and treatment were associated with change or loss of parenting efficacy in mothers (Kuswanto et al., 2018; Tavares et al., 2018), particularly when mothers perceived their children to be emotionally and psychologically affected by their cancer diagnosis (Kuswanto et al., 2018). Consistent with prior research (e.g. Moore et al., 2015), our findings demonstrate that poorer parenting efficacy correlated with more symptoms of depression and stress. Notably, parenting efficacy no longer had an association with any of the psychological distress variables once mothers’ self-compassion was included in the regression models, with both self-warmth and self-coldness emerging as critical predictors of distress.

Fortunately, once mothers’ self-compassion was considered, self-warmth mitigated the negative relationship between parenting efficacy in predicting stress. Our results extended upon previous findings on the role of self-compassion as a buffer to distress during adverse conditions (Abdollahi et al., 2020; Przezdziecki et al., 2013; Todorov et al., 2019) by demonstrating this protective effect in women with breast cancer. As proposed by Costa et al. (2016), self-compassion is involved in affective regulation by balancing the soothing and threat systems. Thus, it is plausible that self-warmth activates the self-soothing system associated with feelings of safeness and effective emotional regulation when facing parenting challenges and deactivates the threat system associated with feelings of insecurity and fears of failure. Therefore, our research further highlights the importance of engaging in activities that encompass self-warmth, such as self-kindness in response to shortfalls, practicing mindfulness and improving a sense of common humanity, as a protective factor against stress for mothers who experience poor parenting efficacy.

Mothers who engaged in more self-coldness (i.e. self-judgement, isolation and over-identification) reported more symptoms of depression, anxiety and stress, regardless of their parenting efficacy. The robustness of the links between self-coldness and symptoms of psychological distress highlights the importance of this negative component of self-compassion and aligns with research finding greater self-compassion associated with less depressive symptoms among lung cancer patients (Siwik et al., 2022) and with fewer depressive and anxiety symptoms in a mixed sample of cancer patients (Wei et al., 2023). Self-coldness can manifest in unhelpful strategies that mothers employ in their parenting roles or more generally, such as over-identifying with, suppressing or avoiding their negative emotions; strategies shown to predict worse psychological symptoms among women receiving radiation therapy for breast cancer (Guimond et al., 2020). Self-coldness may also involve ruminative thinking (or lack of objective perspective) about failures and difficulties, and the tendency to be self-reliant and setting strict standards for themselves when facing parenting challenges and difficulties (Arambasic et al., 2019). Given our findings, decreasing self-coldness in response to perceived shortfalls could possibly reduce psychological distress in mothers with cancer. This may be in the form of providing psychosocial support that encourages mothers to be aware of their rumination and unhelpful strategies, and to promote peer-to-peer support to reduce isolation and foster a sense of community with other mothers who have shared experience of juggling parenting expectations and responsibilities since their cancer diagnosis (Allen and Leary, 2010; Kuswanto et al., 2018).

Aside from self-coldness, child-related factors, rather than parenting efficacy, appeared to be most relevant for anxiety symptoms. Mothers with more children tended to report more anxiety, as did mothers whose children were older. These findings are consistent with prior research, whereby mothers with older children were more distressed than those with younger children, presumably because older children tend to be more cognitively aware of the meaning and repercussions of cancer compared to younger children (Compas et al., 1994; Huizinga et al., 2011; Kissane and Parnes, 2014; Watson et al., 2006), mothers are more willing to share information regarding the nature of cancer with the older children (Compas et al., 1994; Fitch et al., 1999), and because challenges associated with parenting older children may be more emotionally demanding (Arès et al., 2014).

### Study limitations and suggestions for future research

Our data were collected from a self-selected group of women within the BCNA community. These women were more likely to have attained tertiary education and work in paid employment, and the majority were also married and living with their partner or spouse. Therefore, our findings may not be generalisable to mothers with different socio-demographic backgrounds. In addition, the age range of the mothers’ children is very wide. While this may increase generalisability to mothers at different parenting stages, the implications for parenting efficacy should be considered as, developmentally, different ages would be associated with different parenting demands. However parenting efficacy is a perceptions of one’s ability to engage in the behaviours expected in their role as parents generally, such as meeting their child’s emotional needs or their own expectations of themselves, without focus on specific tasks or demands (Jones and Prinz, 2005). Therefore, parenting efficacy is likely relevant across the child’s lifespan despite shifts in the nature of the demands. Accordingly, there was no evidence for an association between child age and parenting efficacy in this sample or in a recent systematic review (Fang et al., 2021). Nevertheless, further investigation of predictors and outcomes of parenting efficacy in specific age ranges, as well as changes in parenting efficacy over time, is recommended.

Having a relatively small sample size also reduced statistical power in our analyses, which can increase the likelihood of type II errors (false negatives) for small effects. Nevertheless, the study was sufficiently powerful to detect moderate effects which are arguably more clinically meaningful. Lastly, the current study uses cross-sectional data, which limits conclusions about causality and alternative explanations must also be considered. For instance, whilst we have presumed that parenting efficacy affects levels of psychological distress, it is also possible that psychological distress leads to poorer parenting efficacy as mothers who experience high levels of distress may be, or perceive themselves to be, less capable in parenting effectively (Fang et al., 2021; Gondoli and Silverberg, 1997). Moreover, mothers’ self-compassion may affect their judgements of their parenting capabilities. For instance, those higher in self-coldness may rate their parenting more harshly, which is consistent with the relationships between parenting efficacy and self-compassion variables revealed in the current research. While the sequencing in the models investigated was conceptually plausible and research informed, longitudinal research would be necessary to verify the directionality of these relationships and presents a promising avenue for future research.

### Clinical implications

Mothers experiencing high levels of psychological distress may benefit from psychosocial support from health care providers. Our findings emphasising the roles of self-warmth and self-coldness, more so than parenting efficacy, in mothers’ psychological distress underscore self-compassion as a promising point of intervention. Self-compassion is a modifiable and trainable characteristic (Arambasic et al., 2019; Neff and Germer, 2013) and there is some evidence of the effectiveness of psychotherapy interventions that incorporate self-compassion in alleviating psychological distress in oncology populations. For example, cancer survivors who participated in mindfulness-based interventions have reported shifts from patterns of disconnection and preoccupation towards slowing down, reduced absorption in negative self-relating, enhanced connections with others and relief from emotional tension (L’Estrange et al., 2016). In addition, a systematic review and meta-analysis provided evidence that parenting interventions that included self-compassion components can increase self-compassion and decrease depression, anxiety and stress among parents over time (Jefferson et al., 2020). Therefore, whilst targeted interventions that improve parenting efficacy may be important, health care professionals should prioritise assessing, monitoring and incorporating self-compassion-based interventions in providing psychosocial support for mothers who are cancer survivors.

The social stigma of cancer (Kim et al., 2012), and beliefs that caring for children is primarily a mother’s responsibility despite their cancer diagnosis, may be potential barriers to mothers exercising compassion towards themselves. This might also be the case for societal and cultural beliefs that reject self-focused attention (L’Estrange et al., 2016). Neff and Pommier (2013) have suggested that women and mothers are more likely to show compassion towards others, which can be attributed to the sex-role socialisation promoting the nurturing and self-sacrificing woman (Neff and Pommier, 2013). Fear of compassion, which comprises fear of self-compassion and fear of compassion from others (Gilbert et al., 2011), has also been highlighted as a potential barrier to self-compassion in both non-clinical and clinical populations. One qualitative study showed that, whilst participants agreed that self-compassion was beneficial for improving mental health, they also believed it would make them vulnerable and that others would judge them (Campion and Glover, 2017). Recent research has also demonstrated that mothers’ fear of self-compassion can elicit negative reactions when imagining themselves engaging in health behaviours, such as feeling like a failure, irresponsible or careless (Simpson et al., 2022). Given the potential benefits to health and wellbeing for mothers who are breast cancer survivors, future research should examine the impact of the social stigma of cancer and fear of compassion to effectively and sensitively address barriers to self-compassion within this population of mothers.

## Conclusion

This is the first study to investigate the interactive relationship between parenting efficacy and self-compassion to understand psychological distress among mothers who are breast cancer survivors. Our findings extended upon the argument that promoting self-compassion may potentially alleviate psychological distress in an oncology population, specifically demonstrating the value of both enhancing self-warmth and minimising self-coldness in mothers who are breast cancer survivors.

## Supplemental Material

sj-docx-1-hpq-10.1177_13591053231222162 – Supplemental material for Self-compassion and parenting efficacy among mothers who are breast cancer survivors: Implications for psychological distress Supplemental material, sj-docx-1-hpq-10.1177_13591053231222162 for Self-compassion and parenting efficacy among mothers who are breast cancer survivors: Implications for psychological distress by Carissa Nadia Kuswanto, Lesley Stafford, Penelope Schofield and Jessica Sharp in Journal of Health Psychology
