# Supplementary material for: Self-compassion and parenting efficacy among mothers who are breast cancer survivors: Implications for psychological distress
Source: J Health Psychol. 2024 Jan 23;29(5):425–37. doi: 10.1177/13591053231222162 (PMC11005311; doi:10.1177/13591053231222162)
Supplement: sj-docx-1-hpq-10.1177_13591053231222162 – Supplemental material for Self-compassion and parenting efficacy among mothers who are breast cancer survivors: Implications for psychological distress [file sj-docx-1-hpq-10.1177_13591053231222162.docx]

**Supplementary Table 1.**

*Demographic and clinical characteristics of mothers in the current study.*

| Demographic variables | Mean | | | SD | | *n* | | % |  |
| --- | --- | --- | --- | --- | --- | --- | --- | --- | --- |
| Age of participants | 50.92 | | | 6.63 | |  | | 34 - 69 |  |
| Age of the oldest child at survey | 21.27 | | | 8.95 | | - | | 7 - 36 |  |
| Age of the youngest child at survey | 17.41 | | | 7.75 | | - | | 4 - 30 |  |
| Number of children |  | | |  | |  | |  |  |
| 1-2 | - | | | - | | 66 | | 69.5% |  |
| ≥ 3 | - | | | - | | 29 | | 30.5% |  |
| Relationship status |  | | |  | |  | |  |  |
| Single | - | | | - | | 12 | | 12.6% |  |
| Married/De facto | - | | | - | | 83 | | 87.4% |  |
| Working full-time/part-time | - | | | - | | 74 | | 77.9% |  |
| Completed tertiary education | - | | | - | | 81 | | 85.3% |  |
| Living arrangement |  | | |  | |  | |  |  |
| With spouse/partner and children | - | | | - | | 68 | | 71.6% |  |
| With children only | - | | | - | | 6 | | 6.3% |  |
| With spouse/partner only | - | | | - | | 12 | | 12.6% |  |
| Alone, or with other relative | - | | | - | | 6 | | 6.3% |  |
| Born outside of Australia | - | | | - | | 16 | | 16.8% |  |
| Clinical variables | Mean | | | SD | | *n* | | % |  |
| Stage of cancer |  | | |  | |  | |  |  |
| Stage O-I | - | | | - | | 45 | | 54.7% |  |
| Stage II | - | | | - | | 36 | | 40.8% |  |
| Stage III-IV | - | | | - | | 14 | | 5.3% |  |
| Months since diagnosis | 77.59 | | | 53.14 | | - | | - |  |
| Months since surgery | 66.17 | | | 46.94 | | - | | - |  |
| Treatment combination |  | | |  | |  | |  |  |
| Surgery only | - | | | - | | 6 | | 6.3% |  |
| Surgery/Hormone | - | | | - | | 4 | | 4.2% |  |
| Surgery/Chemotherapy | - | | | - | | 7 | | 7.4% |  |
| Surgery/Chemotherapy/Hormone | - | | | - | | 13 | | 13.7% |  |
| Surgery/Chemotherapy/Radiotherapy | - | | | - | | 11 | | 11.6% |  |
| Surgery/Radiotherapy/Hormone | - | | | - | | 12 | | 12.6% |  |
| Surgery/ Chemotherapy Radiotherapy/Hormone | - | | | - | | 42 | | 44.2% |  |
| Measures | |  | Mean | | SD | | Possible Range (Observed Range) | | |
| *Depression, Anxiety and Stress Scale (DASS)* | | |  | |  | | 0 – 42 | | |
| DASS-D (Depression) | |  | 6.48 | | 7.89 | | (0 – 38) | | |
| DASS-A (Anxiety) | |  | 4.36 | | 5.97 | | (0 – 28) | | |
| DASS-S (Stress) | |  | 10.93 | | 8.04 | | (0 – 32) | | |
| *Cancer-related Parenting Self Efficacy (CaPSE)* | |  |  | |  | |  | | |
| *Parenting efficacy* | | | 99.47 | | 11.19 | | 24 – 144  (76 – 120) | | |
| *Self-Compassion Scale (SCS)* | | |  | |  | | 1 – 5 | | |
| Self-warmth | |  | 3.18 | | .69 | | (1.70 – 4.50) | | |
| Self-coldness | |  | 2.75 | | .91 | | (1.13 – 4.48) | | |

*N* = 95

**Supplementary Table 2**

*Correlations for Model Variables and Key Demographic and Clinical variables*

| Variable | 1 | 2 | 3 | 4 | 5 | 6 |
| --- | --- | --- | --- | --- | --- | --- |
| 1. Depression | - |  |  |  |  |  |
| 2. Anxiety | .49^***^ | - |  |  |  |  |
| 3. Stress | .56^***^ | .67^***^ | - |  |  |  |
| 4. Parenting efficacy | -.28^***^ | -.13 | -.35^***^ | - |  |  |
| 5. Self-warmth | -.25^***^ | -.17 | -.34^***^ | .22^*^ | - |  |
| 6. Self-coldness | .50^***^ | .36^***^ | .59^***^ | -.37^*^ | -.49^***^ | - |
| 7. Age of mother | .10 | .23^*^ | .13 | -.16 | .13 | .11 |
| 8. Age of youngest child | .19 | .33^***^ | .17 | -.17 | .05 | .15 |
| 9. Number of children | -.04 | .25^*^ | .07 | .01 | .03 | -.24^*^ |
| 10. Relationship status ^a^ | -.07 | .01 | -.10 | .15 | -.08 | -.04 |
| 11. Employment status ^b^ | -.15 | -.21^*^ | -.16 | -.05 | .02 | -.11 |
| 12. Education status ^c^ | -.11 | .02 | .06 | .05 | .09 | -.07 |
| 13. Time since diagnosis | .18 | .22^*^ | .16 | -.18 | -.05 | .18 |
| 14. Cancer stage ^d^ | -.11 | -.19 | -.08 | .02 | .15 | -.14 |
| 15. Time since surgery | .04 | -.06 | -.02 | -.13 | -.08 | .12 |
| 16. Chemotherapy ^e^ | -.13 | .10 | -.02 | .22^*^ | .13 | -.05 |
| 17. Radiotherapy ^f^ | .01 | .09 | .03 | -.06 | -.00 | .07 |
| 18. Hormone therapy ^g^ | -.16 | -.18 | -.11 | .05 | .12 | -.09 |

Note:

^a^ Without a partner: 0, with a partner: 1

^b^ Unemployed: 0, Working parttime/fulltime: 1

^c^ Did not complete tertiary education: 0, Completed tertiary education: 1

^d^ Stage O-I: 0, Stage II-IV: 1

^e^ Did not have chemotherapy: 0, Did have chemotherapy: 1

^f^ Did not have radiotherapy: 0, Did have radiotherapy: 1

^g^ Did not have hormone therapy: 0, Did have hormone therapy: 1

**p* < 0.05, ****p* < 0.01
